# Supplementary material for: Digital RNA sequencing using unique molecular identifiers enables ultrasensitive RNA mutation analysis
Source: Commun Biol. 2024 Mar 1;7:249. doi: 10.1038/s42003-024-05955-7 (PMC10907754; doi:10.1038/s42003-024-05955-7)
Supplement: Supplementary file 2 — Supplementary Information [file 42003_2024_5955_MOESM2_ESM.pdf]

Supplementary Figures

Supplementary Figure 1. The concept of UMIs.

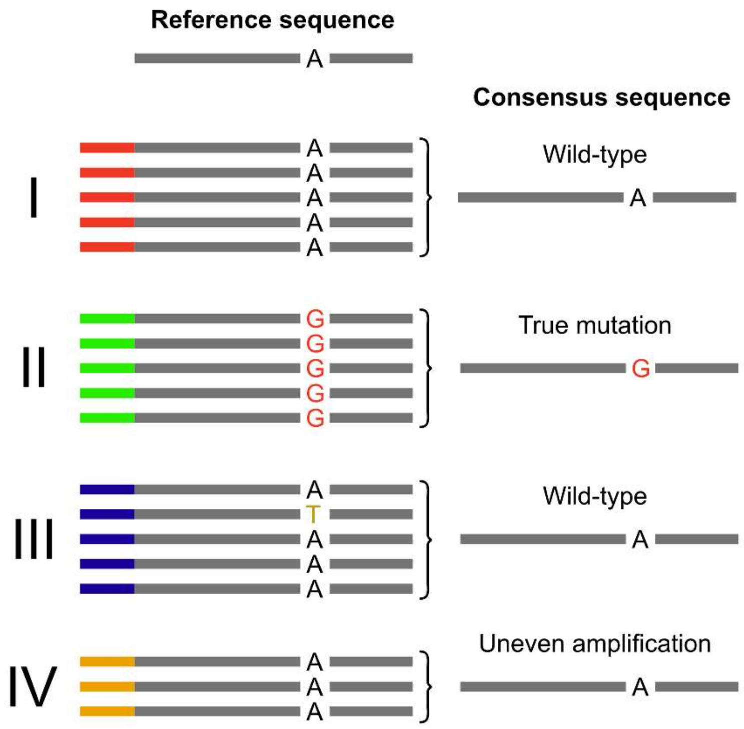

After sequencing, all molecules originating from the same initial molecule (I-IV) are labeled with identical UMIs (red, green, blue and yellow). For every target sequence, reads with identical UMIs can be bioinformatically collapsed into one consensus read per UMI. To call a mutation, the majority of all reads with the same identical UMI also need to have identical allele variant. Hence, molecules with true mutations (molecule II) can be identified and technical errors (molecule III) will be corrected. The use of UMIs will also help reduce quantification biases since all reads with the same identical UMI are collapsed into a single consensus read. For example, molecules I and IV are both generating one consensus read each, despite having different number of reads per UMI.

Supplementary Figure 2. UMI tagging during barcoding PCR.

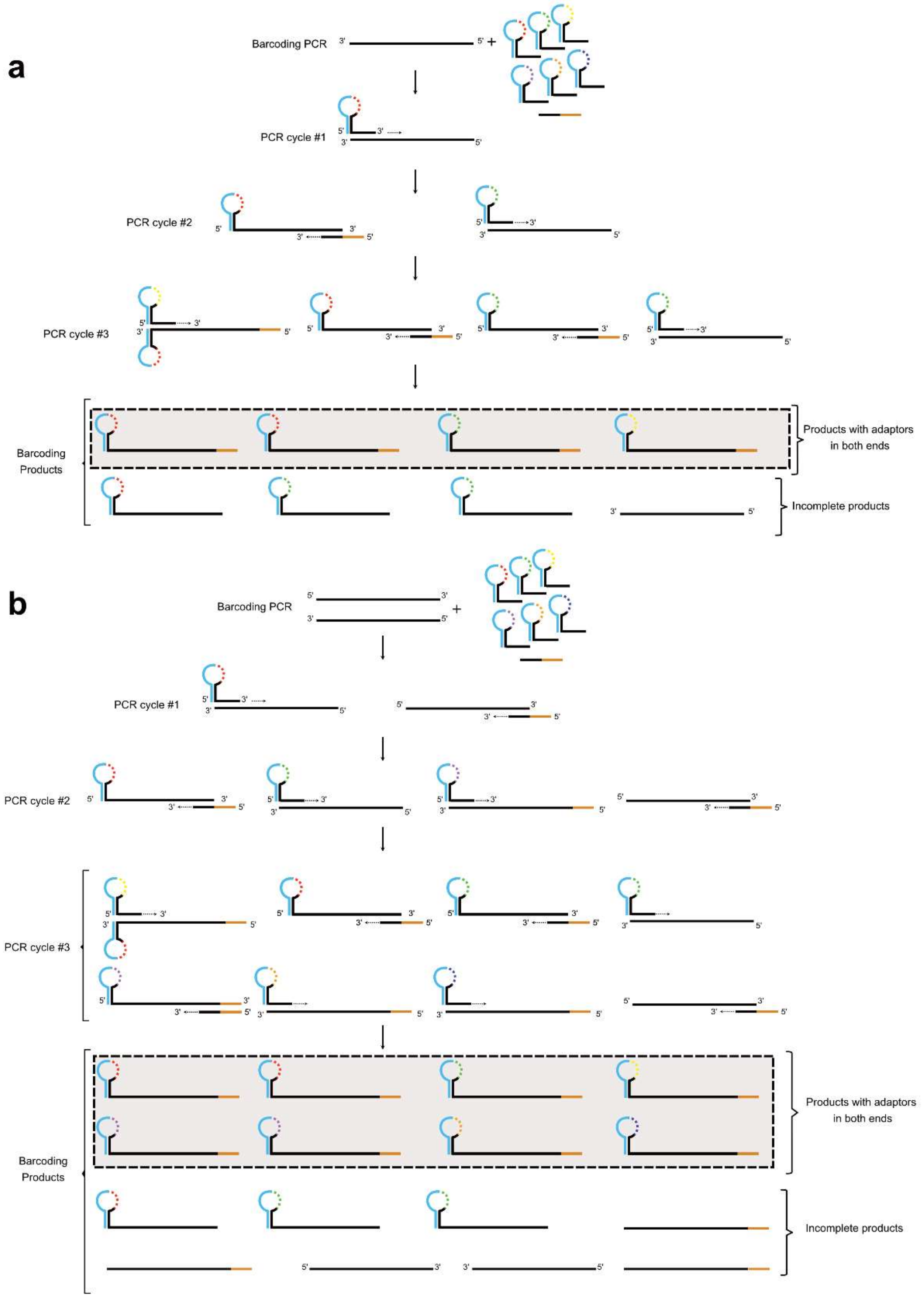

a) Barcoding PCR using single-stranded complementary DNA as input. The output includes three uniquely barcoded molecules. **b)** Barcoding PCR using double-stranded genomic DNA as input. The output includes six uniquely barcoded molecules.

Supplementary Figure 3. UMI handling and bioinformatics.

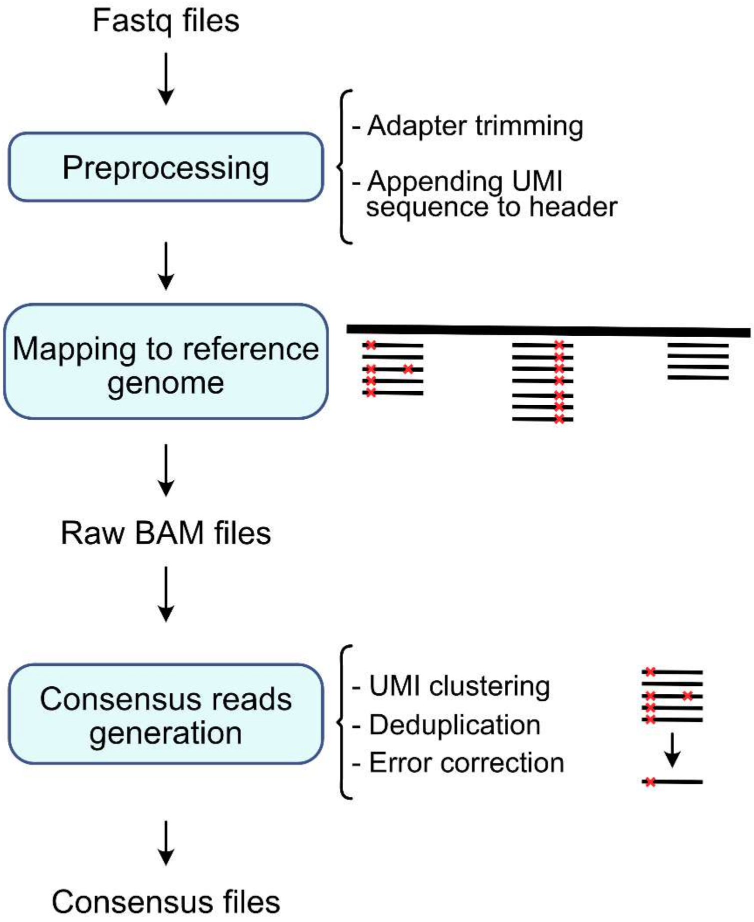

Schematic overview of bioinformatics pipeline using UMIErrorCorrect [28]. This Python package consists of three subprocesses: fastq data preprocessing, mapping to reference genome and consensus reads generation.

Supplementary Figure 4. Assay positions in *TP53* and *HBB* genes.

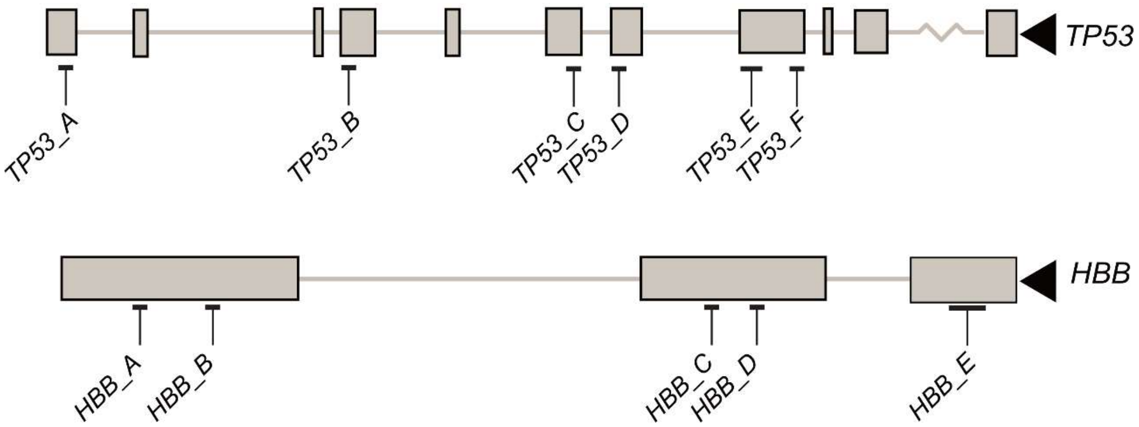

The *TP53* assays cover commonly mutated nucleotide positions, while the *HBB* has no known recurrent mutations.

Supplementary Figure 5. UMI-error correction.

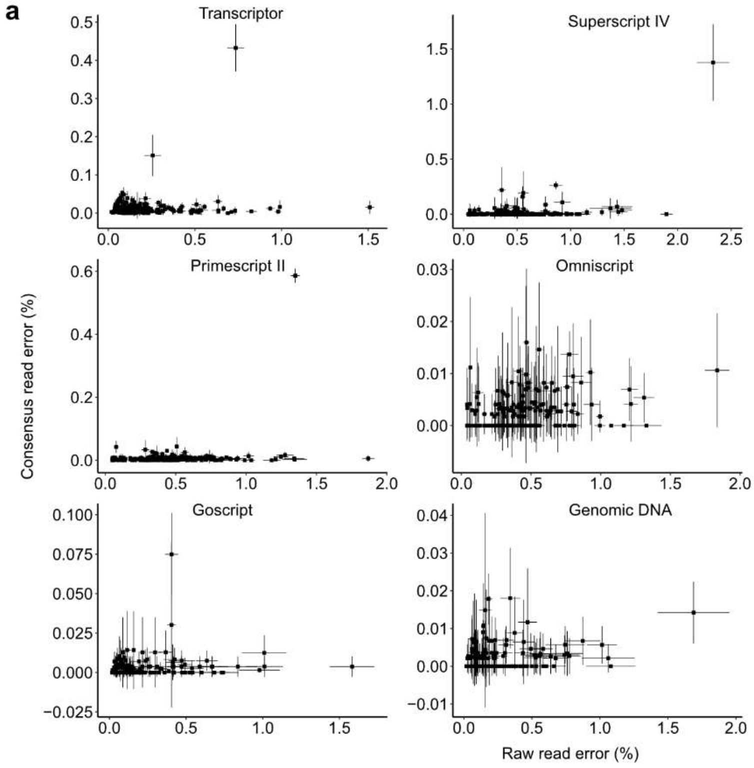

RT condition	A	C	G	T	D	I
Transcriptor	0	1.67	0	19775	71.33	11
Superscript IV	0	0	0	2844	3	36.67
Sensiscript	0	0	0	1261.67	0	0
Primescript II	0	0	0.33	13546	3	76
Omniscript	0	0	0	12103	1.33	0.33
Grandscript	0	0.33	0	8402.33	3	68.67
Goscript	0	0	0	13052.33	5	4.67
Genomic DNA	0	0	0	9944.33	0	0

a) Error rates before and after UMI-correction are shown for each nucleotide position. Mean ± SD is shown, n = 3. **b)** Outlier data. Nucleotide position 7669645 in *TP53* displayed high error rates before and after UMI-error correction for a subset of RT conditions. The number of consensus reads for each nucleotide base type (A, C, G and T) is shown as well as the number of deletions (D) and insertions (I). Mean values are shown, n = 3.

Supplementary Figure 6. Sequencing errors in relation to sequence context.

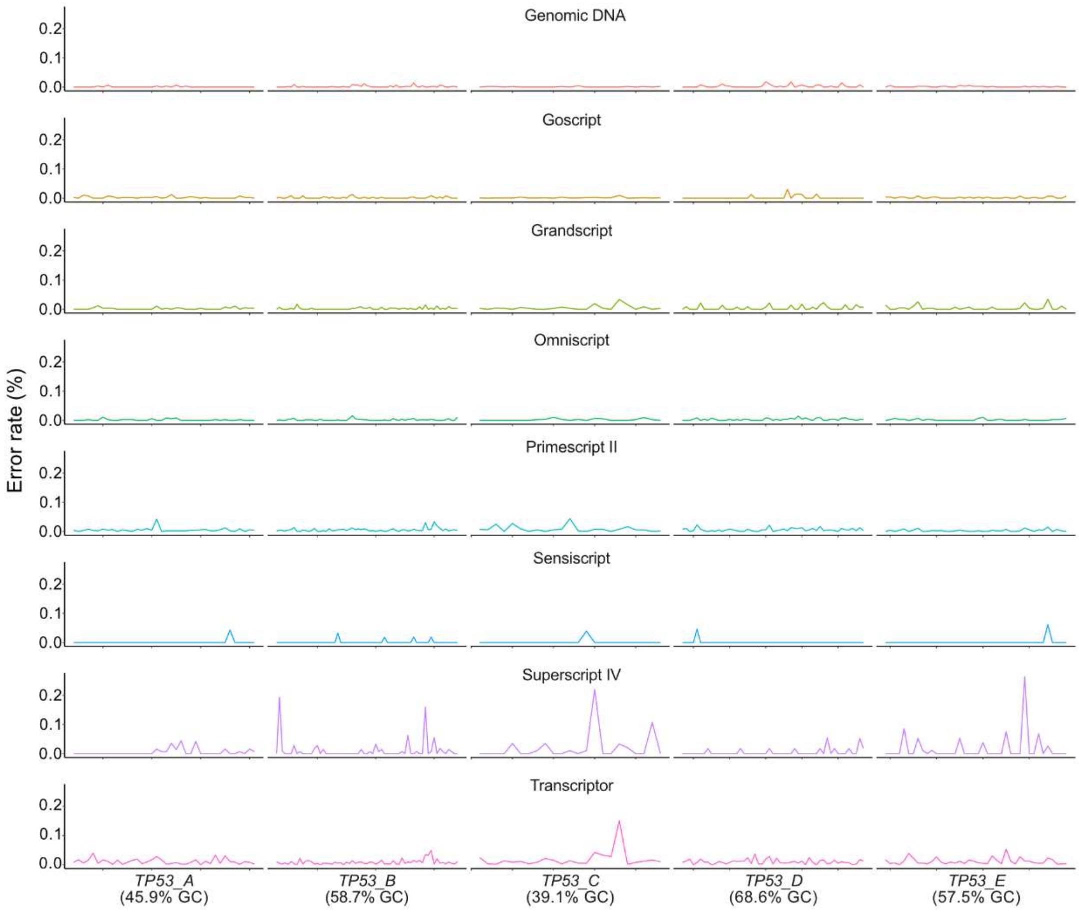

The mean error frequency per nucleotide position after UMI correction for all reverse transcription condition in *TP53* is shown, n = 3. The outlier nucleotide position 7669645 was excluded for visualization purpose.

Supplementary Figure 7. Complementary DNA yield.

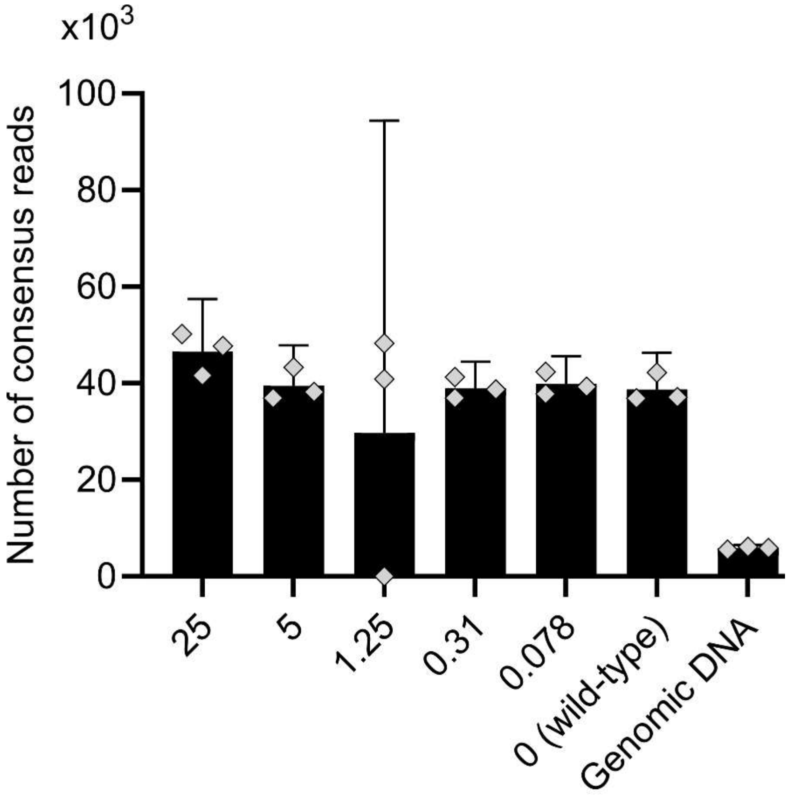

Number of consensus reads for *TP53* assay F, analyzing 200 ng total RNA. Ten nanograms genomic DNA was analyzed as control. The expected spike-in mutant allele frequency is shown at the x-axis. Mean ± 95% CI is shown, n = 3.

Supplementary Figure 8. Detection of *TP53* mutation with and without UMI correction.

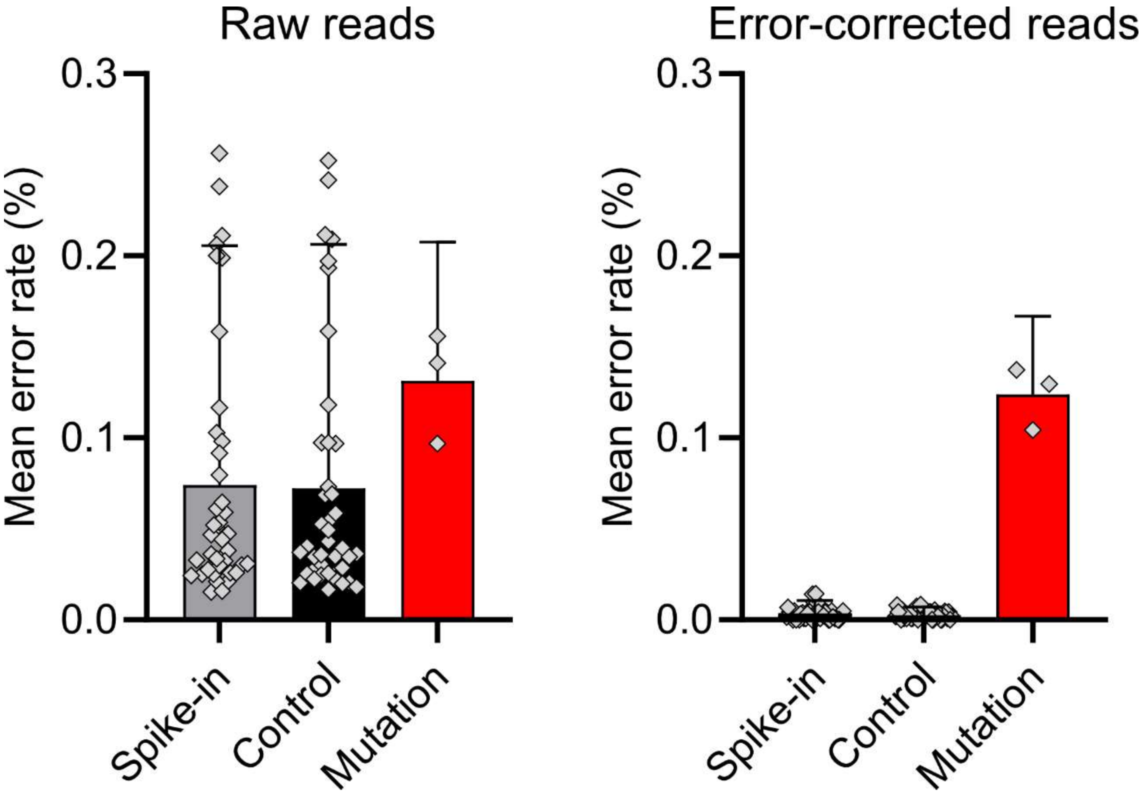

The mean error rates for samples with (gray) and without (black) spike-in mutant molecules are shown, excluding the nucleotide position with mutation. Mean and 95% confidence of distribution is shown (Mean + 1.96 SD, n = 40-41). Mean + 95% CI is shown for the mutated nucleotide position (red), n = 3.

Supplementary Figure 9. Breast cancer panel performance.

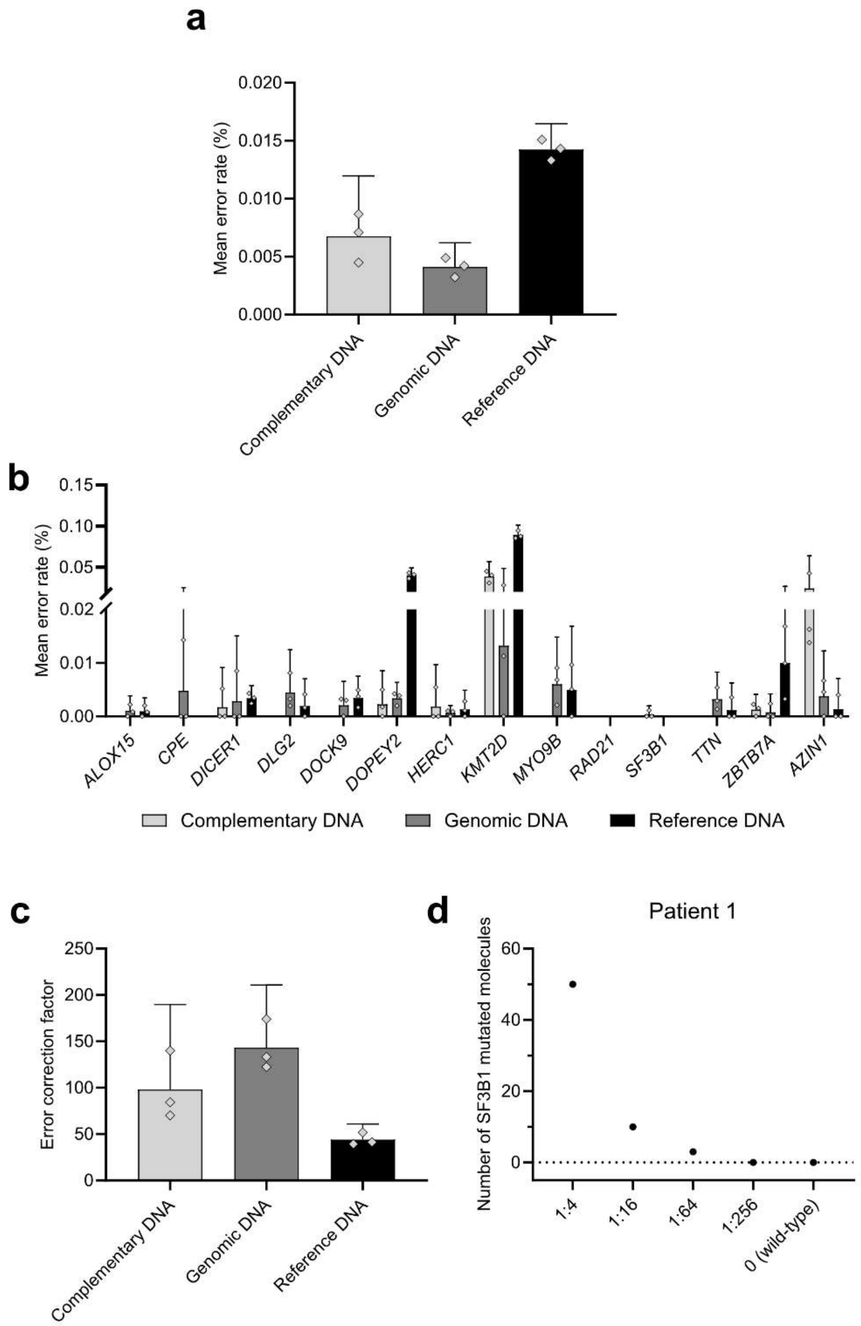

a) Mean error rates across all assays. The error rate was calculated as the total number of non-reference reads divided by the total number of detected reads per nucleotide position using consensus reads. The errors were then averaged for all nucleotide positions and assays. Mean ± 95% CI is shown, n = 3. **b)** Error rate per assay. Mean ± 95% CI is shown, n = 3. **c)** Error correction factor using UMIs. The error rates before and after using UMIs were calculated and compared. Mean ± 95% CI is shown, n = 3. **d)** Detection of mutations in diluted RNA samples. The number of mutated *SF3B1* molecules is shown when the total RNA from patient 1 was diluted with total RNA from MLS 1765-92 cells, keeping the amount of total RNA constant at 200 ng in RT, n = 1. In the 1:64 dilution sample, three molecules were observed, while no molecules were detected in the 1:256 dilution sample as well as in the control sample containing only MLS 1765-92 total RNA.

Supplementary Figure 10. ***HBB*** **error rates.**

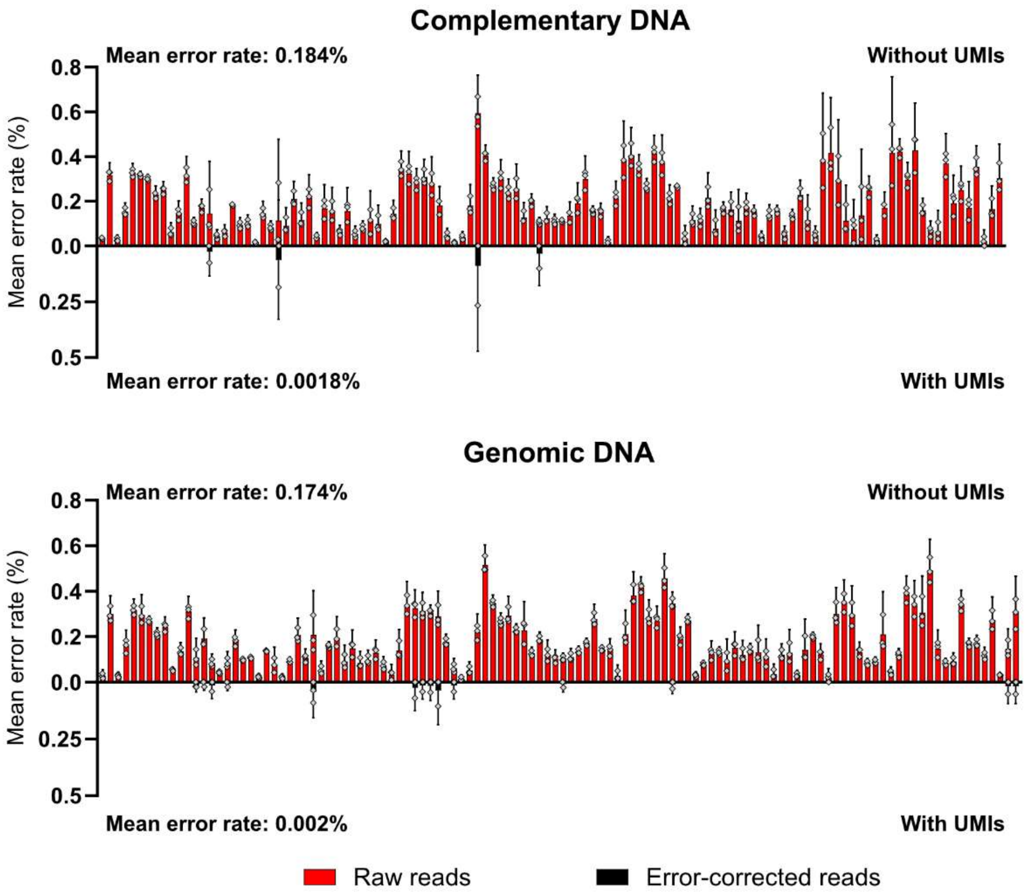

HBB data across all five assays with and without error correction using UMIs. The error rate per position was calculated as the total number of non-reference reads divided by the total number of detected reads with and without UMI-error correction. Mean + 95% CI is shown, n = 3. The individual samples are not shown for nucleotide position with only zeros.
