# Supplementary material for: Digital RNA sequencing using unique molecular identifiers enables ultrasensitive RNA mutation analysis
Source: Commun Biol. 2024 Mar 1;7:249. doi: 10.1038/s42003-024-05955-7 (PMC10907754; doi:10.1038/s42003-024-05955-7)
Supplement: Supplementary file 3 — Description of Additional Supplementary Files [file 42003_2024_5955_MOESM3_ESM.docx]

Description of Additional Supplementary Files

**File name:** Supplementary Data 1

**Description:** Number of molecules used in Figure 2.

**File name:** Supplementary Data 2

**Description:** Number of molecules used in Figure 3d

**File name:** Supplementary Data 3

**Description:** Barcoding PCR primers.

**File name:** Supplementary Data 4

**Description:** Number of raw reads.

**File name:** Supplementary Data 5

**Description:** The source data behind the graphs in the paper

**File name:** Supplementary Data 6

**Description:** The source data behind the graphs in the supplementary

File name: Supplementary Data 4 Description: Number of raw reads. File name: Supplementary Data 5 Description: The source data behind the graphs in the paper File name: Supplementary Data 6 Description: The source data behind the graphs in the supplement
