# Supplementary material for: Digital RNA sequencing using unique molecular identifiers enables ultrasensitive RNA mutation analysis
Source: Commun Biol. 2024 Mar 1;7:249. doi: 10.1038/s42003-024-05955-7 (PMC10907754; doi:10.1038/s42003-024-05955-7)
Supplement: Supplementary file 10 — Reporting Summary [file 42003_2024_5955_MOESM10_ESM.pdf]

nature portfolio

Corresponding author(s): Anders Ståhlberg

Last updated by author(s): Jan 30, 2024

Reporting Summary

Nature Portfolio wishes to improve the reproducibility of the work that we publish. This form provides structure for consistency and transparency in reporting. For further information on Nature Portfolio policies, see our Editorial Policies and the Editorial Policy Checklist.

Statistics

For all statistical analyses, confirm that the following items are present in the figure legend, table legend, main text, or Methods section.

n/a	Confirmed	
☐	☒	The exact sample size (*n*) for each experimental group/condition, given as a discrete number and unit of measurement
☐	☒	A statement on whether measurements were taken from distinct samples or whether the same sample was measured repeatedly
☐	☒	The statistical test(s) used AND whether they are one- or two-sided<br>*Only common tests should be described solely by name; describe more complex techniques in the Methods section.*
☒	☐	A description of all covariates tested
☒	☐	A description of any assumptions or corrections, such as tests of normality and adjustment for multiple comparisons
☐	☒	A full description of the statistical parameters including central tendency (e.g. means) or other basic estimates (e.g. regression coefficient) AND variation (e.g. standard deviation) or associated estimates of uncertainty (e.g. confidence intervals)
☒	☐	For null hypothesis testing, the test statistic (e.g. *F*, *t*, *r*) with confidence intervals, effect sizes, degrees of freedom and *P* value noted<br>*Give P values as exact values whenever suitable.*
☒	☐	For Bayesian analysis, information on the choice of priors and Markov chain Monte Carlo settings
☒	☐	For hierarchical and complex designs, identification of the appropriate level for tests and full reporting of outcomes
☒	☐	Estimates of effect sizes (e.g. Cohen's *d*, Pearson's *r*), indicating how they were calculated

Our web collection on statistics for biologists contains articles on many of the points above.

Software and code

Policy information about availability of computer code

Data collection: n/a

Data analysis: Digital error correction was performed using umierrorcorrect v0.29, publicly available at https://github.com/stahlberggroup/umierrorcorrect

For manuscripts utilizing custom algorithms or software that are central to the research but not yet described in published literature, software must be made available to editors and reviewers. We strongly encourage code deposition in a community repository (e.g. GitHub). See the Nature Portfolio guidelines for submitting code & software for further information.

Data

Policy information about availability of data

All manuscripts must include a data availability statement. This statement should provide the following information, where applicable:

- Accession codes, unique identifiers, or web links for publicly available datasets
- A description of any restrictions on data availability
- For clinical datasets or third party data, please ensure that the statement adheres to our policy

Raw sequencing data in FASTQ format will be available at the NCBI Sequence Read Archive under submission ID PRJNA967219.

Human research participants

Policy information about studies involving human research participants and Sex and Gender in Research.

Reporting on sex and gender	Female for breast cancer samples
Population characteristics	n/a
Recruitment	Primary invasive breast carcinomas collected at surgery for biomarker analysis. Patients were not recruited for this study.
Ethics oversight	Medical Faculty Research Ethics Committee (# S164-02, Gothenburg, Sweden)

Note that full information on the approval of the study protocol must also be provided in the manuscript.

Field-specific reporting

Please select the one below that is the best fit for your research. If you are not sure, read the appropriate sections before making your selection.

☒ Life sciences ☐ Behavioural & social sciences ☐ Ecological, evolutionary & environmental sciences

For a reference copy of the document with all sections, see nature.com/documents/nr-reporting-summary-flat.pdf

Life sciences study design

All studies must disclose on these points even when the disclosure is negative.

Sample size	n = 3, enable parametric comparisons between different experimental parameters.
Data exclusions	One technical outlier was removed in the experiments evaluating dynamic range using different RNA dilutions (Fig 3D).
Replication	Technical replicates, n=3.
Randomization	n/a
Blinding	n/a

Reporting for specific materials, systems and methods

We require information from authors about some types of materials, experimental systems and methods used in many studies. Here, indicate whether each material, system or method listed is relevant to your study. If you are not sure if a list item applies to your research, read the appropriate section before selecting a response.

Materials & experimental systems

n/a	Involved in the study	
☒	☐	Antibodies
☐	☒	Eukaryotic cell lines
☒	☐	Palaeontology and archaeology
☒	☐	Animals and other organisms
☒	☐	Clinical data
☒	☐	Dual use research of concern

Methods

n/a	Involved in the study	
☒	☐	ChIP-seq
☒	☐	Flow cytometry
☒	☐	MRI-based neuroimaging

Eukaryotic cell lines

Policy information about cell lines and Sex and Gender in Research

Cell line source(s)	Non-commercial myxoid liposarcoma cell lines were derived and provided by Professor Pierre Åman for research only.
Authentication	Validated by detection of cell line specific fusion oncogenes at RNA level and short tandem repeat analysis performed by Eurofins (Used cell lines do not have a match).
Mycoplasma contamination	The cell lines tested negative for mycoplasma contamination.

Commonly misidentified lines<br>(See ICLAC register)	None. The used cell lines are characterized by fusion oncogenes that are not present in any other available cell lines.
